# Insights Into Visual Rehabilitation: Pan-Retinal Photocoagulation for Proliferative Diabetic Retinopathy

**DOI:** 10.7759/cureus.54273

**Published:** 2024-02-15

**Authors:** Meghavi Pandya, Shashank Banait, Sachin Daigavane

**Affiliations:** 1 Ophthalmology, Jawaharlal Nehru Medical College, Datta Meghe Institute of Higher Education & Research, Wardha, IND

**Keywords:** patient education, laser technology, diabetic retinopathy management, visual rehabilitation, pan retinal photocoagulation, proliferative diabetic retinopathy

## Abstract

This review comprehensively explores pan-retinal photocoagulation (PRP) as a pivotal intervention in visually rehabilitating individuals afflicted with proliferative diabetic retinopathy (PDR). The review begins by elucidating the significance of PDR within the spectrum of diabetic retinopathy (DR), emphasizing the progressive nature of the disease and the consequential impact on visual health. A detailed analysis of PRP follows, encompassing its definition, purpose, and historical development, shedding light on the procedural intricacies and mechanisms of action. The postoperative care and follow-up section underscores the necessity of vigilant monitoring for complications, visual recovery, and the importance of regular ophthalmic check-ups. The subsequent discussion delves into patient education and counseling, stressing the need to manage expectations, encourage lifestyle modifications, and highlight the significance of follow-up appointments. The review concludes with insights into future directions, including advancements in laser technology and emerging therapies, offering a glimpse into the evolving landscape of DR management. By addressing ongoing challenges and embracing innovative approaches, this review provides a comprehensive guide for clinicians, researchers, and healthcare practitioners who visually rehabilitate individuals struggling with PDR.

## Introduction and background

Diabetic retinopathy (DR) stands as a prevalent and potentially sight-threatening complication of diabetes mellitus, affecting a substantial portion of the global population. As a progressive disease, DR evolves through various stages, with proliferative diabetic retinopathy (PDR) marking an advanced and critical phase characterized by the growth of abnormal blood vessels on the retina. Understanding the intricate nature of PDR is pivotal, given its substantial impact on visual health and overall quality of life for individuals struggling with diabetes [1]. DR, a microvascular complication stemming from chronic hyperglycemia, manifests as damage to the blood vessels nourishing the retina. The disease progression involves distinct stages, beginning with non-proliferative stages and culminating in the more severe and sight-threatening PDR. A comprehensive grasp of the underlying pathophysiology of DR is imperative for contextualizing the significance of PDR within the broader spectrum of diabetic eye complications [2].

PDR marks the advanced stage of DR, where the retinal vasculature becomes increasingly compromised, leading to the formation of abnormal new blood vessels or neovascularization. These fragile vessels are prone to leakage, hemorrhage, and retinal detachment. The consequences of PDR extend beyond the ocular realm, as the associated vision loss significantly impacts the daily lives of affected individuals. Recognizing the significance of PDR is fundamental for implementing timely interventions that can halt its progression and mitigate visual impairment [3]. Visual impairment resulting from PDR not only diminishes the ability to perform routine tasks but also poses considerable challenges to maintaining an independent and fulfilling lifestyle. The need for effective visual rehabilitation strategies in PDR is underscored by the potential for mitigating vision loss, enhancing functional vision, and improving the overall well-being of affected individuals. Addressing this imperative requires a multifaceted approach, with PRP emerging as a cornerstone in the visual rehabilitation arsenal [4].

This review aims to comprehensively examine pan-retinal photocoagulation (PRP) as a crucial intervention in visually rehabilitating individuals with PDR. By delving into the procedural aspects, indications, outcomes, and associated challenges, this review offers insights that contribute to a nuanced understanding of PRP's role in managing PDR and fostering visual recovery. Through a synthesis of current literature and research findings, the review aims to guide clinicians, researchers, and healthcare practitioners in optimizing visual outcomes for patients struggling with this sight-threatening complication of diabetes.

## Review

Understanding PRP

PRP is a therapeutic laser procedure designed to treat PDR by utilizing controlled laser burns to induce selective photocoagulation of the peripheral retina. The primary purpose of PRP is to reduce neovascularization, the hallmark of PDR, thereby mitigating the risk of complications such as vitreous hemorrhage and retinal detachment. By strategically applying laser burns to the entire peripheral retina, PRP aims to create a barrier that limits the growth of abnormal blood vessels, preserving retinal function and preventing vision loss [5]. The mechanism of action underlying PRP involves the selective application of laser energy to coagulate and photocoagulate areas of the peripheral retina. The laser energy is absorbed by pigmented cells in the retinal pigment epithelium, leading to thermal coagulation of the targeted tissue. This process induces a controlled tissue reaction that results in the closure of abnormal blood vessels, reducing the risk of hemorrhage and other complications associated with PDR [6]. Furthermore, PRP induces a reduction in the oxygen demand of the peripheral retina, prompting a decrease in the production of vascular endothelial growth factor (VEGF). VEGF is a critical factor in the development of neovascularization. By modulating VEGF levels and promoting the regression of abnormal vessels, PRP contributes to stabilizing the retinal microenvironment.

Indications for PRP

Diagnostic Criteria for PDR

Neovascularization: Identifying neovascularization, whether on the optic disc (NVD) or elsewhere on the retina (NVE), is a pivotal diagnostic criterion for PDR. Neovascularization indicates the development of abnormal, fragile blood vessels with an increased propensity for leakage and hemorrhage. Recognizing neovascularization is crucial, as it marks an advanced stage of diabetic retinopathy where the risk of vision-threatening complications is significantly heightened. Early detection of neovascularization is imperative for timely intervention and initiating appropriate treatments such as the PRP [7].

Vitreous hemorrhage: The occurrence of vitreous hemorrhage, often stemming from neovascularization, is a compelling indication for implementing PRP. Vitreous hemorrhage involves bleeding into the vitreous humor, potentially impairing vision and causing significant visual disturbances. PRP is recommended to address the underlying neovascularization responsible for the bleeding. By halting the abnormal blood vessel growth through laser therapy, PRP aims to prevent recurrent vitreous hemorrhages, ultimately safeguarding and preserving visual function in individuals with PDR [8].

Tractional retinal detachment: Tractional retinal detachment, a severe complication of PDR, arises from the presence of fibrous tissue exerting tractional forces on the retina. Neovascularization is crucial to developing these fibrous adhesions, necessitating intervention to prevent or manage tractional retinal detachment. In such cases, PRP is indicated to address and reduce neovascularization, thereby mitigating the forces pulling on the retina. By targeting the underlying pathology, PRP plays a crucial role in preventing the progression of tractional retinal detachment and preserving the structural integrity of the retina [9].

Identification of High-Risk Characteristics

Extensive neovascularization: The degree of neovascularization, especially when it spans a substantial area of the retina, is a critical indicator of heightened risk for complications. Extensive neovascularization implies a more advanced stage of DR, with a greater potential for adverse outcomes. In such cases, PRP is frequently recommended as an intervention strategy. Through laser therapy, PRP addresses widespread neovascularization, aiming to halt its progression and mitigate the associated risks of vision-threatening complications [10].

Presence of high-risk neovascularization: Neovascularization at specific locations, such as the optic disc (NVD) or anterior to the equator of the eye, is considered high-risk due to its association with an elevated likelihood of vitreous hemorrhage and retinal detachment. Identifying such high-risk neovascularization is crucial in determining the urgency and intensity of intervention. PRP emerges as a recommended approach in these cases, strategically employed to target and manage the high-risk neovascularization, ultimately reducing the potential for severe complications and preserving visual function [11].

Rapid disease progression: Patients exhibiting accelerated progression from non-proliferative stages to PDR represent a subgroup at higher risk for severe complications. The rapid advancement of the disease necessitates timely and proactive intervention to manage its aggressive course effectively. In such instances, PRP is a crucial therapeutic measure that can help impede further progression and reduce the risk of complications. By intervening promptly, PRP becomes a cornerstone in the management of diabetic retinopathy, particularly in cases of rapid disease progression, contributing to the preservation of visual health [12].

Role in Preventing Vision Loss

Stabilization of abnormal blood vessels: A primary mechanism of action for PRP is the induction of photocoagulation in abnormal blood vessels within the retina. This strategic laser therapy aims to stabilize the existing neovascularization, preventing their further growth. By arresting the progression of these abnormal blood vessels, PRP significantly reduces the risk of complications such as hemorrhage and retinal detachment. This intervention plays a pivotal role in maintaining the structural integrity of the retina, ultimately contributing to the preservation of visual function in individuals with PDR [6].

Reduction of vitreous hemorrhage: PRP effectively minimizes the occurrence of vitreous hemorrhage, a common consequence of leaky neovascularization. Through targeted laser therapy, PRP seals these abnormal blood vessels, preventing leakage and reducing the risk of bleeding into the vitreous humor. By addressing the underlying pathology contributing to vitreous hemorrhage, PRP plays a crucial role in preserving visual function and minimizing the disruptive impact of bleeding on visual acuity [13].

Prevention of tractional retinal detachment: One of the sight-threatening complications associated with PDR is tractional retinal detachment, resulting from fibrous tissue formation and tractional forces. PRP intervenes by addressing the neovascularization that contributes to the development of these fibrous adhesions. Through targeted laser therapy, PRP mitigates the risk of tractional retinal detachment by reducing the tractional forces exerted on the retina. This proactive approach is integral in preventing severe structural damage to the retina and underscores PRP's role in safeguarding visual health in individuals with advanced diabetic retinopathy [14].

Preoperative assessment and preparation

Patient Evaluation

Visual acuity: The preoperative PRP assessment includes a meticulous visual acuity evaluation. This fundamental measure establishes a baseline for the patient's visual function, offering insights into the extent of impairment caused by PDR. Visual acuity assessment is integral to understanding the impact of the disease on the patient's ability to perceive and interpret visual stimuli. This baseline information aids clinicians in tailoring the PRP intervention to address specific visual challenges, ultimately contributing to the overall management strategy [15].

Fundus examination: A comprehensive fundus examination is imperative in the preoperative evaluation for PRP. This thorough assessment allows clinicians to identify specific characteristics of PDR, including neovascularization, hemorrhages, and any signs of retinal detachment. Fundus findings are critical factors guiding the decision-making process regarding the extent and localization of laser application during PRP. By identifying and characterizing the pathologies in the fundus, healthcare providers can strategically plan laser therapy to target and mitigate the underlying abnormalities associated with PDR [16].

Imaging studies: Incorporating imaging studies, such as fluorescein angiography and optical coherence tomography (OCT), into the preoperative evaluation provides detailed insights into the retinal vasculature. These imaging modalities reveal areas of leakage, ischemia, and neovascularization, offering valuable information for treatment planning. Fluorescein angiography highlights blood flow dynamics, while OCT provides high-resolution cross-sectional retina images. These imaging studies aid clinicians in identifying high-risk regions that may require focused laser therapy during PRP. The information gleaned from these studies enhances the precision and efficacy of the PRP intervention, contributing to the overall success of the treatment strategy [17].

Considerations for Special Populations

Pregnant women: For pregnant women, undergoing PRP necessitates special considerations due to the exposure to laser energy. Given the potential risks to the developing fetus, a thorough discussion about these concerns is paramount. The procedure may be deferred or modified based on the gestational age, with careful consideration of the potential impact on the pregnancy. This delicate balance between the clinical necessity of PRP and the well-being of the unborn child underscores the importance of individualized decision-making and open communication between the healthcare provider and the expectant mother [18].

Pediatric patients: In the case of pediatric patients, a tailored approach is often required, recognizing the unique challenges associated with their ability to cooperate during the procedure. Sometimes, sedation or anesthesia might be considered to ensure patient comfort and compliance. This consideration aims to create an environment conducive to successful PRP while prioritizing the well-being of the pediatric patient. The individualized nature of this approach emphasizes the importance of adapting the procedure to the specific needs and capacities of the pediatric population [19].

Elderly patients: Elderly individuals may present with coexisting medical conditions or reduced tolerance to certain aspects of the PRP procedure. Therefore, a comprehensive assessment of overall health is essential to identify and mitigate potential complications. This assessment may involve a review of medical history, medication profiles, and pre-existing ocular conditions. Adjustments to the PRP procedure or additional monitoring may be implemented based on the specific health status of the elderly patient, ensuring a safe and effective intervention tailored to their individual needs [20].

Patients with systemic comorbidities: Patients with systemic comorbidities, such as cardiovascular disease or renal impairment, require meticulous attention and coordination with their primary care team. Close collaboration is essential to optimize their overall health before undergoing PRP. Precautions may be necessary during the procedure to accommodate the specific medical needs and potential vulnerabilities associated with systemic comorbidities. This interdisciplinary approach aims to enhance the safety and efficacy of PRP while addressing the broader health concerns of the patient [21].

Patients with previous ocular surgeries: Patients with a history of previous ocular surgeries present unique challenges due to potential alterations in ocular anatomy. Careful consideration is crucial in determining the feasibility and safety of PRP in such cases. Reviewing prior surgical records and conducting imaging studies become valuable tools for assessing the ocular landscape and planning laser therapy accordingly. This attention to detail ensures that the PRP procedure is tailored to the specific ocular characteristics of individuals with a history of previous ocular surgeries [22].

Patients with limited mobility or cognitive impairment: Individuals with limited mobility or cognitive impairment may require additional support and accommodations during the PRP procedure. Creating a comfortable and accommodating environment is crucial for a successful session. This may involve optimizing the physical setup, assisting with positioning, and ensuring clear communication with the patient. The emphasis is on fostering an inclusive and supportive environment that addresses the unique needs of individuals with limited mobility or cognitive impairment during the PRP procedure [23].

The procedure of PRP

Laser Settings and Parameters

The PRP procedure involves using a laser to treat retinal ischemia and neovascularization, particularly in PDR. The laser settings and parameters for PRP typically include [24,25]: using a standard argon-type laser, settings that involve burns ranging approximately 200μ to 500μ in size, pulse durations of 100 milliseconds, and power of 200-250 mW, The goal is to produce burns that are grey and to avoid dense white burns. Adjustment of the laser settings can help minimize patient discomfort, for example, by reducing the duration of the pulse from 100 msec [26]. The selection of laser setting parameters depends on the treatment goals, the clarity of the ocular media, and the fundus pigmentation [24]. It is essential to apply PRP around the periphery of the retina, and the laser does not apply all of the spots of a pattern at once; instead, each is delivered over the set time [27].

Treatment Planning

The treatment planning for PRP involves using a special laser to make tiny burns that seal the retina and stop vessels from growing and leaking. PRP is typically recommended for individuals diagnosed with proliferative retinopathy, particularly in the advanced stage of retinopathy, where abnormal new blood vessels may rupture and bleed inside the eye. PRP aims to prevent the development of new vessels over the retina and elsewhere, not to regain lost vision. The initial treatment usually consists of approximately 1,500-2,000 spots of laser per eye, which may be done in one or more sessions. After the treatment, vision may be blurred, and patients are advised to have someone drive them home and to relax for the rest of the day. Most patients can resume activities within a few days, and regular follow-up visits are required. It's important to note that there is no improvement in vision after the laser treatment, and vision may decrease due to edema/swelling of the retina. However, it may improve to its previous level in time [28,29].

Techniques for Laser Application

Direct vs indirect laser delivery: Laser application techniques in PRP include direct and indirect laser delivery methods. The direct method involves using a slit lamp system, where the laser is attached to the typical ophthalmic slit lamp, and the laser energy is delivered in a coaxial fashion. The patient is placed in a seated position, and the chin is placed on the chin rest. On the other hand, the indirect method utilizes an indirect laser ophthalmoscope (headlamp/BIO) to focus the laser light onto the retina. This method requires expertise and is typically performed or supervised by an experienced ophthalmologist to avoid technical complications [30]. The choice of technique depends on the patient's specific requirements and the ophthalmologist's expertise. Both methods have effectively treated PDR and other retinal neovascularization conditions. PRP aims to prevent the development of new vessels over the retina and elsewhere, although it may not restore lost vision. It is important to note that severe complications with PRP are infrequent. Still, like any surgical procedure, it does have risks, which can be minimized by seeking treatment from a specialist experienced in pan-retinal photocoagulation [28].

Spot size and density: The spot size and density for PRP typically depend on the treatment goals and the clarity of the ocular media. The laser spot size for PRP is typically 200-500 μm, and the laser power is adjusted to achieve grey or light cream-colored burns. The goal is to create grey burns and avoid dense white burns. The initial treatment usually consists of approximately 1,500-2,000 spots of laser per eye, which may be done in one or more sessions. The macula may be kept in view while the mid periphery of the retina is being treated, making these lenses ideal for PRP. High-plus-power lenses provide a spot size that is magnified over the laser setting size, and the magnification factor depends on the lens used. The selection of laser setting parameters depends on the treatment goals, the clarity of the ocular media, and the fundus pigmentation. Adjusting the laser settings can assist in minimizing patient discomfort, for example, by reducing the pulse duration from 100 msec [24,26,28].

Pain Management During the Procedure

Pain management during PRP is crucial to ensure patient comfort and reduce stress during the procedure. There are several options for pain relief during PRP, including [31,32]. Retrobulbar anesthesia injects an anesthetic into the tissue surrounding the eye to numb the area. Peribulbar anesthesia injects an anesthetic into the tissue surrounding the eye to numb the area. Sub-tenon anesthesia injecting an anesthetic under the conjunctiva to numb the area.

Topical anesthetics: Applying eye drops containing anesthetic to the eye provides relief. Oral pain relief cocktail administering an oral medication to help relieve pain during the procedure. A study evaluated the efficacy of an oral pain relief combination during PRP for DR and found that it effectively reduced pain [32]. However, the majority of people (73%) still experienced moderate or more significant pain during PRP [31]. It is essential to discuss pain management options with your ophthalmologist to determine the most appropriate method for your specific situation.

Postoperative care and follow-up

Monitoring for Complications

Vitreous hemorrhage: Emphasizing the importance of regular fundus examinations is critical to proactively managing potential complications, particularly vitreous hemorrhage, following treatment. Vigilant monitoring enables clinicians to promptly detect any signs of bleeding within the vitreous, a complication that may arise postoperatively. Immediate intervention may be necessary in such cases, underscoring the significance of regular fundus examinations in preventing and addressing complications associated with vitreous hemorrhage [33].

Macular edema: Monitoring patients for the development of macular edema is a crucial aspect of postoperative care, particularly in those with pre-existing diabetic macular edema. Implementing optical coherence tomography (OCT) proves invaluable in assessing macular thickness and identifying early signs of edema. This diagnostic tool facilitates the timely detection of macular changes, allowing for proactive intervention to manage and mitigate the progression of edema, ultimately preserving central vision [34].

Visual field changes: Given the proximity of treated areas to the peripheral retina, regular evaluation for changes in visual field function is imperative. Visual field testing is a valuable diagnostic tool to detect adverse effects on the patient's peripheral vision. Incorporating this assessment into regular follow-ups ensures the comprehensive monitoring of visual function, enabling clinicians to promptly identify and address any visual field changes [35].

Intraocular pressure: The routine intraocular pressure measurement is essential to postoperative care. Regular monitoring is vital as an elevation in intraocular pressure may occur following the intervention. Timely measurement and assessment enable healthcare providers to identify deviations from the norm and intervene promptly to prevent complications associated with increased intraocular pressure, promoting optimal ocular health in the postoperative period [36].

Visual Recovery and Rehabilitation

Patient education: Ensuring patients comprehensively understand the postoperative journey is paramount. Communicating realistic expectations, including the possibility of transient visual changes and the gradual nature of visual improvement following the intervention, is essential. Emphasizing the importance of strict compliance with postoperative care instructions is crucial for optimal outcomes. Educating patients about the expected trajectory of visual changes post-surgery enhances their preparedness and fosters a cooperative approach to recovery [37].

Rehabilitation services: Integrating rehabilitation services becomes a valuable consideration for individuals facing significant visual impairment before or after the operation. In cases where visual changes persist postoperatively, rehabilitation services such as low vision aids or vision therapy may be recommended. These services enhance functional vision, address specific challenges, and optimize visual capabilities. By incorporating rehabilitation into the postoperative plan, healthcare providers aim to improve overall visual outcomes and support patients in adapting to changes in their vision effectively [38].

Adaptive strategies: Encouraging adaptive strategies is essential, particularly for patients struggling with changes in peripheral vision. Training in eccentric viewing involves using a different part of the retina for visual tasks, and orientation and mobility techniques can be beneficial, especially when visual field deficits are present. By empowering patients with adaptive strategies, they can effectively cope with changes in their visual perception and maintain a sense of independence. This proactive approach to adaptive strategies aligns with a patient-centered care model, addressing individual needs and enhancing overall visual well-being [39].

Importance of Regular Follow-Up

Assessment of treatment efficacy: The significance of follow-up appointments lies in the thorough assessment of PRP efficacy. During these appointments, clinicians meticulously evaluate the regression of neovascularization, gauging the success of the intervention in halting abnormal blood vessel growth within the retina. Additionally, preventing vision-threatening complications is scrutinized, providing a comprehensive understanding of the treatment's impact on preserving visual health. These assessments serve as pivotal benchmarks in guiding further management and ensuring the ongoing efficacy of the PRP intervention [40].

Early detection of recurrence: Vigilant monitoring for any signs of disease recurrence constitutes a vital aspect of follow-up appointments. Early detection of subtle changes in the retina enables prompt intervention, a crucial measure in preventing the progression of proliferative diabetic retinopathy. By identifying signs of recurrence early, clinicians can proactively tailor interventions, potentially mitigating the risk of vision-threatening complications. This proactive approach underscores the preventive nature of follow-up assessments in the comprehensive management of DR [41].

Adjustment of treatment plan: The dynamic nature of DR necessitates flexibility in treatment plans. Follow-up appointments offer a platform for clinicians to assess the patient's response to PRP and identify any emerging complications. Depending on these evaluations, adjustments to the treatment plan may be required. This may involve additional laser sessions to address persisting neovascularization or incorporating alternative therapeutic modalities to optimize outcomes. The adaptability of the treatment plan underscores the personalized and responsive nature of DR management [42].

Patient education: Regular follow-up appointments are invaluable opportunities for ongoing patient education. Clinicians reinforce vital aspects of diabetic control, emphasizing the critical role of lifestyle modifications and the importance of adhering to prescribed medications. Healthcare providers empower individuals to actively participate in their care by continuously educating patients during follow-ups. This educational component contributes to long-term adherence to preventive measures, promoting overall ocular health and facilitating a collaborative approach to managing DR [43].

Efficacy and outcomes

Reduction of Retinal Neovascularization

PRP effectively reduces the incidence of iris neovascularization (NVI) in various ischemic ocular conditions. A study reported that PRP effectively reduced the incidence of neovascular glaucoma in patients with NVI, with 65% of patients showing regression of NVI after PRP [44]. Additionally, PRP has been widely used to treat neovascular proliferative diseases secondary to conditions such as sickle cell retinopathy and venous occlusive diseases, further demonstrating its efficacy in reducing retinal neovascularization [45]. Furthermore, PRP combined with intravitreal anti-VEGF agents has shown positive outcomes in treating high-risk PDR, as evidenced by various systematic reviews and meta-analyses [46]. While PRP is effective, it is essential to be aware of potential complications associated with the procedure, such as choroidal effusions, exudative retinal detachments, and macular edema [25].

Prevention of Vision Loss

PRP has effectively reduced the risk of severe vision loss in patients with PDR. However, it is essential to note that while PRP effectively reduces the risk of severe vision loss, it can lead to visual field changes and potential visual function effects [47]. A study highlighted the long-term visual function effects of PRP, indicating that the functional results achieved are compatible with an active life for most patients, allowing them to meet the legal criteria for driving [48]. Additionally, research has been conducted to assess the effects of diabetic retinopathy and PRP on photoreceptor cell function, aiming to understand better the complex pathophysiology of vision loss in DR [49]. It is essential to closely monitor the visual outcomes of PRP and consider further management strategies for patients with PDR, even after achieving stability [48]. While PRP has been shown to reduce the risk of severe vision loss by 50%, it is essential to be aware of potential complications associated with the procedure, such as choroidal effusions, exudative retinal detachments, and macular edema [26].

Long-Term Visual Rehabilitation

PRP has effectively reduced the risk of severe vision loss in patients with PDR. However, monitoring the long-term visual rehabilitation outcomes is essential, as PRP can lead to visual field changes and potential visual function effects. A study found that the functional results achieved in patients undergoing bilateral PRP were compatible with an active life, allowing most patients to meet the legal criteria for driving, which is related to visual field requirements [50]. Another study demonstrated that despite achieving PDR stability, most patients lost clinically meaningful vision [48]. A post hoc analysis of a randomized clinical trial evaluating changes in visual field throughout five years among eyes treated with PRP or ranibizumab found that PRP caused visual field losses, and these losses accumulated between 2 and 5 years such that by five years, the visual field losses were significant [47]. While PRP effectively reduces the risk of severe vision loss, it is crucial to closely monitor the long-term visual rehabilitation outcomes and consider further management strategies for patients with PDR, even after achieving stability.

Complications and adverse effects

Immediate Complications

Immediate PRP complications may include discomfort during treatment, which can be managed with pain relief options such as retrobulbar anesthesia, peribulbar anesthesia, sub-tenon anesthesia, topical anesthetics, or oral pain relief cocktails. However, serious complications associated with PRP are rare but can include choroidal effusions, exudative retinal detachments, macular edema, visual field deficits, and night vision defects [25,51]. These complications are closely tied to laser parameters such as increased duration, which causes increased dispersion of thermal energy within the retina and choroid [25]. The advent of newer laser delivery systems, such as the multispot pattern laser, has significantly mitigated but has yet to eliminate these issues [25]. It is essential to discuss the potential risks and benefits of PRP with your ophthalmologist to determine the most appropriate treatment plan for your specific situation.

Delayed Complications

Choroidal effusions refer to fluid accumulation in the space between the retina and the choroid, which may cause a decline in vision [25]. Exudative retinal detachments involve the build-up of fluid beneath the retina, leading to the separation of the retina from the underlying tissue and the potential for vision loss [25]. Macular edema is characterized by swelling in the central retina due to fluid accumulation, resulting in diminished vision and distortions in vision perception [25]. Visual field defects denote permanent alterations in the visual field resulting from laser treatment, contributing to a decrease in vision quality [25]. Night vision defects, difficulty seeing in low-light conditions, can arise from laser treatment and associated retinal changes [25]. It is imperative to diligently monitor patients for potential delayed complications and address them promptly. Regular follow-up visits and dilated eye exams play a critical role in the early detection and management of these complications. Although serious complications are rare, their occurrence can significantly impact a patient's vision and overall quality of life [52].

Strategies for Minimizing Adverse Effects

Several strategies can be employed to enhance the safety and efficacy of laser treatments, specifically in the context of photocoagulation. Firstly, careful attention to laser settings, including pulse duration, power, and spot size adjustments, is crucial. These adjustments minimize patient discomfort and play a pivotal role in reducing the risk of complications associated with the procedure. Another approach involves dividing the PRP treatment into multiple sessions. This step can effectively decrease the likelihood of adverse effects, as highlighted in existing literature [26]. Ensuring proper patient selection is equally essential; verifying that individuals undergoing PRP are suitable candidates and have well-controlled underlying conditions, such as diabetes, can significantly lower the risk of complications [26].

Preoperative preparation is an essential component of risk reduction. Patients should be instructed to abstain from blood-thinning medications and alcohol before the procedure to mitigate potential complications [26]. Following the treatment, comprehensive postoperative care is crucial. This includes providing detailed instructions to patients regarding activity restrictions and follow-up visit schedules. Such measures contribute not only to optimal patient recovery but also to the early detection of potential complications [26]. Regular follow-up visits are imperative for continuously monitoring patients who have undergone PRP. Dilated eye exams during these visits are critical for the early detection and management of complications such as choroidal effusions, exudative retinal detachments, macular edema, visual field defects, and night vision defects [26].

Comparative approaches

Pan-Retinal Photocoagulation vs. Anti-VEGF Therapy

PRP and anti-vascular endothelial growth factor (anti-VEGF) therapy stand as effective treatments for PDR. The decision between the two approaches involves weighing the pros and cons, emphasizing the need for an individualized choice based on diverse ocular and patient factors [53]. PRP has been a longstanding treatment for PDR [53]. It effectively reduces oxygen demand in the retina and prevents new vessel development [46]. However, PRP has drawbacks, potentially leading to visual field deficits and night vision defects [40]. On the other hand, anti-VEGF therapy involves the intravitreal injection of anti-VEGF agents like bevacizumab, ranibizumab, and aflibercept [40]. Inhibition of VEGF has emerged as a crucial aspect of anti-neovascularization therapy [40]. Anti-VEGF therapy offers practicality and cost-effectiveness, often resulting in better visual acuity outcomes and lower incidences of vitreous hemorrhage [54]. A systematic review and meta-analysis revealed that the combination of PRP and intravitreal anti-VEGF agents exhibits superior efficacy in delaying vision loss associated with DR and reducing the number of vessels [46]. Despite this, comprehensive evidence regarding the long-term impact of anti-VEGF compared to PRP and studies involving larger sample sizes is crucial for a more nuanced understanding [54]. The choice between PRP and anti-VEGF therapy hinges on various factors, including the patient's ocular health, diabetes control, and personal preferences. Ongoing trials and research endeavors are anticipated to contribute more information, facilitating an improved understanding of the optimal treatment approach for PDR [53]. The dynamic landscape of DR management calls for a nuanced evaluation of each patient's unique circumstances to make informed decisions that balance therapeutic efficacy and potential adverse effects.

Combination Therapies

Combination therapies for PDR have been explored to improve treatment outcomes and reduce the risk of complications. One study investigated the combination of intravitreal administration of Ozurdex (a dexamethasone-based drug) followed by individually selected laser coagulation, which is effective against various retinal conditions [55]. Another study examined the combination of PRP and intravitreal anti-VEGF injections, showing similar efficacy in treating PDR [56]. The combination of PRP and anti-VEGF therapy has been suggested to provide better visual acuity outcomes and lower vitreous hemorrhage [57]. Combination therapies for PDR have been explored to improve treatment outcomes and reduce the risk of complications. The combination of PRP and intravitreal anti-VEGF injections has been found to have similar efficacy in treating PDR [30]. Other combination therapies, such as the combination of intravitreal administration of Ozurdex followed by laser coagulation, have also shown effectiveness against various retinal conditions [55]. However, more research is needed to determine the optimal combination therapy approach for PDR, considering individual patient factors and each treatment option's potential benefits and risks.

Patient education and counseling

Managing Expectations

Explanation of PRP: Providing a lucid and succinct overview of PRP is fundamental in ensuring patients' understanding of this critical procedure. Emphasis is placed on the primary purpose of PRP, which is to halt the progression of PDR. Patients are informed about the intended outcomes, elucidating the role of PRP in reducing abnormal blood vessel growth within the retina. The goal is to prevent vision-threatening complications, fostering a comprehensive comprehension of the procedure's significance in preserving visual health [58].

A realistic timeline for visual recovery: Transparent communication regarding the gradual nature of visual recovery post-PRP is essential in managing patient expectations. Patients are guided through a realistic timeline, emphasizing that improvement occurs gradually over time. It is crucial to convey that transient visual changes may be experienced before the full benefits of the treatment manifest. This emphasis on the temporal aspect of visual recovery aims to instill patience and understanding, fostering a cooperative approach between healthcare providers and patients as they navigate the post-PRP period [59].

Possibility of residual vision changes: Acknowledging the potential for residual vision changes, particularly in peripheral vision, is critical to patient education. Open communication about the potential outcomes of PRP treatment allows patients to be prepared for possible visual field deficits. In this context, discussing adaptive strategies and rehabilitation options becomes paramount, providing patients with a proactive approach to address any challenges that may arise post-PRP. This comprehensive discussion ensures that patients are well-informed and empowered to navigate potential residual changes in their vision [60].

Lifestyle Modifications

Diabetes management: Underscoring the pivotal role of optimal diabetes management is paramount in curbing the progression of diabetic retinopathy. Patients are urged to recognize the centrality of adhering to prescribed medication regimens, maintaining consistently healthy blood sugar levels, and embracing a lifestyle that aligns with diabetes-friendly practices. The emphasis on these fundamental aspects of diabetes management is a proactive measure to mitigate the risk of exacerbating retinal complications, reinforcing the interconnected relationship between systemic health and ocular well-being [61].

Blood pressure control: Shedding light on the critical connection between blood pressure control and retinal health is essential. Hypertension poses a significant risk of exacerbating complications related to DR. Patients are informed about the tangible benefits of maintaining blood pressure within a specified target range. By prioritizing blood pressure control, individuals with diabetes contribute significantly to preserving retinal health, recognizing the synergistic impact of systemic factors on ocular complications [62].

Smoking cessation: In the pursuit of comprehensive DR risk mitigation, patients are strongly advised to embark on the journey of smoking cessation. Smoking, identified as a modifiable risk factor, is intricately linked to the progression of DR. Healthcare providers offer resources and unwavering support for those seeking assistance in quitting smoking, recognizing the pivotal role of this lifestyle modification in breaking the chain of exacerbating retinal complications. This tangible and impactful intervention emphasizes empowering individuals to take charge of their ocular health [63].

Regular exercise and healthy diet: Promoting a holistic lifestyle incorporating regular exercise and a balanced diet is a cornerstone in diabetic retinopathy management. Patients are encouraged to embrace physical activity and nutrition as components of overall well-being, recognizing their positive impact on systemic health, including ocular health. By adopting a healthy lifestyle, individuals contribute proactively to preventing complications associated with DR, reinforcing the concept of a holistic approach to health that extends beyond managing diabetes alone [64].

Importance of Regular Ophthalmic Check-ups

Monitoring DR progression: Highlighting regular ophthalmic check-ups is paramount in proactively managing DR. Emphasizing the necessity for periodic assessments is a critical strategy for monitoring the progression of this condition. Regular examinations detect subtle changes in the retina, facilitating timely intervention and mitigating the risk of advancing to more severe stages, notably PDR. This emphasis on vigilant monitoring underscores the proactive approach essential for preserving visual health in individuals with diabetes [65].

Compliance with follow-up appointments: Reinforcing the significance of attending scheduled follow-up appointments constitutes a cornerstone in DR care. Communicating the importance of consistent follow-up, even in the absence of noticeable vision changes, underscores the proactive and preventive nature of regular monitoring. By adhering to scheduled appointments, patients enable healthcare professionals to conduct thorough evaluations, detect emerging issues promptly, and intervene as needed. This emphasis on compliance is a collaborative effort between patients and healthcare providers to ensure the continuous monitoring and management of DR [66].

Ocular health beyond vision: Elevating patient awareness about the comprehensive nature of ophthalmic check-ups extends beyond the assessment of visual acuity. Regular examinations encompass a thorough evaluation of the eyes' overall health, including a meticulous examination of the retina, optic nerve, and other vital structures. This holistic approach provides a comprehensive assessment of ocular health, allowing for the early detection of conditions that may not manifest through changes in vision alone. This broader perspective on ocular health underscores the integral role of regular eye examinations in preserving overall eye function and well-being [67].

Educating on signs of complications: Educating patients about potential signs of complications is a crucial aspect of DR management. Educating individuals about symptoms such as sudden changes in vision, floaters, or flashes of light enables them to recognize warning signs promptly. Stressing the importance of promptly reporting unusual symptoms ensures early intervention and minimizes the risk of severe complications. This educational initiative reinforces a proactive and collaborative approach, where patients play an active role in their eye health by recognizing and communicating potential concerns to their healthcare providers [68].

Future directions and innovations

Advancements in Laser Technology

Selective laser photocoagulation: Advancements in laser technology have led to the exploration of selective laser photocoagulation, a revolutionary approach allowing for precise targeting of specific retinal structures while minimizing collateral damage to adjacent tissues. This technique represents a significant leap forward in the safety and efficacy of laser treatments for retinal disorders. By minimizing unintended effects on healthy tissue, selective laser photocoagulation can enhance treatment outcomes and reduce the risk of adverse effects, presenting a promising avenue for refining laser therapies in ophthalmic practice [69].

Subthreshold laser therapy: The investigation into subthreshold laser therapy introduces a novel technique utilizing laser energy below the threshold of detectable tissue damage. This cutting-edge approach seeks therapeutic benefits while minimizing visible laser scars, addressing a notable concern in traditional laser treatments. By operating below the threshold of tissue damage, subthreshold laser therapy not only offers a discreet alternative but also holds the potential to improve patient comfort and satisfaction. The exploration of this technique underscores a commitment to refining the aesthetics and patient experience in laser-based interventions [70].

Integration of imaging technologies: The integration of advanced imaging technologies, such as adaptive optics and artificial intelligence-assisted imaging, represents a forward-looking initiative to enhance the precision of laser treatments. By leveraging these technologies, clinicians can achieve a more detailed and real-time visualization of retinal structures, enabling accurate targeting during laser procedures. This integration improves treatment efficacy and contributes to a more comprehensive understanding of the retinal microenvironment. Exploring this interdisciplinary approach showcases a commitment to harnessing technological innovations for patient care [71].

Portable laser devices: Innovations in laser technology extend to developing portable and user-friendly laser devices for at-home monitoring and early intervention. This progressive concept aims to empower patients in their care by facilitating regular self-assessments and enabling the delivery of targeted laser treatments under healthcare professionals' guidance. The potential benefits include enhanced patient engagement, timely intervention, and improved overall management of retinal conditions. The exploration of portable laser devices aligns with the evolving landscape of patient-centered care, emphasizing the role of patients as active participants in their treatment regimens [72].

Emerging Therapies in DR

Anti-angiogenic therapies: The advancement of anti-angiogenic therapies represents a dynamic frontier in DR research. This line of investigation revolves around the ongoing evolution of therapies designed to inhibit angiogenesis, particularly those focusing on novel drugs targeting VEGF. Researchers are delving into the intricacies of these new drugs, seeking to understand their mechanisms of action and potential benefits in halting abnormal blood vessel growth in the retina. Moreover, there is a concerted effort to explore sustained-release drug delivery systems, aiming to extend the duration of therapeutic effects and consequently reduce the frequency of injections. The goal is to enhance patient compliance by offering a more convenient and tolerable treatment regimen [73].

Gene therapy: The exploration of gene therapy as a targeted intervention for DR reflects a paradigm shift in treatment strategies. Researchers are conducting a thorough assessment of the potential of gene therapy to modulate gene expression associated with retinal vascular changes. The focus is on understanding how gene-based interventions can be precisely tailored to prevent or even reverse the progression of DR. Safety and efficacy are at the forefront of this exploration, with a keen interest in the molecular mechanisms through which gene therapy may exert its therapeutic effects. This avenue holds considerable promise in providing a more personalized and specific approach to tackling the complex pathophysiology of DR [74].

Neuroprotective agents: A novel dimension in DR research involves the exploration of neuroprotective agents. This area of study is directed towards preserving retinal function and preventing neuronal damage, recognizing the importance of addressing neurodegenerative aspects in addition to vascular components. Researchers are actively investigating compounds that target specific pathways involved in neurodegeneration. The intention is not only to complement existing treatments that predominantly focus on vascular aspects but also to mitigate the impact of neuronal damage, thereby potentially enhancing overall visual outcomes for individuals with DR [75].

Stem cell therapy: The investigation into stem cell therapy represents a frontier of regenerative medicine with profound implications for DR management. Researchers are scrutinizing the application of stem cells for retinal regeneration and repair, exploring the potential of these cells to differentiate into various retinal cell types. The primary aim is to replace damaged cells and restore functional integrity in the diabetic retina. This cutting-edge approach holds promise for addressing the structural deficits associated with DR, offering a novel avenue for therapeutic intervention [76].

Combination therapies: Recognizing the multifaceted nature of DR, researchers are exploring the synergistic effects of combining different therapeutic modalities. This includes investigating the interplay between laser therapy and pharmacological interventions to determine whether a multimodal approach can achieve superior outcomes. The emphasis is on targeting multiple aspects of the complex pathophysiology of DR simultaneously. By combining treatments, researchers aim to optimize efficacy, potentially enhancing the overall success of therapeutic interventions and providing a more comprehensive strategy for managing this intricate and progressive disease [77].

## Conclusions

In conclusion, the comprehensive examination of PRP for PDR provides valuable insights into the intricate landscape of managing this advanced complication of diabetes. We have delved into the significance of PDR, elucidated the purpose and mechanism of PRP, and explored the procedural intricacies, postoperative care considerations, and the vital role of patient education. The implications for clinical practice underscore the need for precision in treatment application, patient-centric postoperative care, and holistic education. As we look toward the future, advancements in laser technology and emerging therapies present promising avenues for refining DR management. Yet, challenges persist, emphasizing the importance of collaborative, multidisciplinary care that prioritizes patient needs. By addressing these ongoing challenges and embracing innovative approaches, clinicians can continue to evolve their strategies, ensuring that individuals with PDR receive optimal care beyond immediate clinical interventions fostering a holistic approach to visual rehabilitation.
